# Prevalence of anxiety and depressive symptoms among obese pregnant and postpartum women: an intervention study

**DOI:** 10.1186/1471-2458-10-766

**Published:** 2010-12-16

**Authors:** Ing-Marie Claesson, Ann Josefsson, Gunilla Sydsjö

**Affiliations:** 1 Linköping University, Faculty of Health Sciences, Division of Obstetrics and Gynaecology, Department of Clinical and Experimental Medicine, S-581 85 Linköping, Sweden

## Abstract

**Background:**

Although studies have shown an association between anxiety and depression and obesity, psychological health among obese women during and after pregnancy has not been carefully studied. The aim of this study was to investigate psychological well-being using symptoms of depression and/or anxiety among obese pregnant women attending a weight gain restriction program and to then compare this group with a control group receiving traditional antenatal care.

**Methods:**

151 obese pregnant women in an intervention group and 188 obese pregnant women in a control group answered the Beck Anxiety Inventory (BAI) and the Edinburgh Postnatal Depression Scale (EPDS). Group differences between the two groups were estimated by using the χ^2 ^- test on categorical variables. The Student's *t*-test on continuous, normally distributed variables measuring changes in mean score on BAI and EPDS over time was used. To make a more comprehensive assessment of group differences, between as well as within the two groups, logistic regressions were performed with the BAI and EPDS as dependent variables, measured at gestational weeks 15 and 35 and 11 weeks postnatal. The grouping variable has been adjusted for socio-demographic variables and complications.

**Results:**

The prevalence of symptoms of anxiety during pregnancy varied between 24% and 25% in the intervention group and 22% and 23% in the control group. The prevalence of symptoms of anxiety postnatally was 9% in the intervention group and 11% in the control group. Five percent of the women in the intervention group and 4% of the women in the control group showed symptoms of anxiety during the course of pregnancy and at the postpartum assessment. The prevalence of symptoms of depression during pregnancy varied between 19% and 22% in the intervention group but was constant at 18% in the control group. Postnatal prevalence was 11% in both groups. Six percent of the women in the intervention group and 4% in the control group showed symptoms of depression during the course of pregnancy and at the postpartum assessment. We found no differences between the two groups as concerns demographic characteristics, weight gain in kg, or the distribution of scores on anxiety and depressive symptoms nor did we find differences in the fluctuation of anxiety and depressive symptoms over time between the women in the intervention group and in the control group.

**Conclusion:**

Obese pregnant women attending an intervention program seem to have the same risk of experiencing anxiety and/or depressive symptoms as do obese pregnant and postnatal women in general.

## Background

Obesity has been reported to have a negative impact on physical health and psychological well-being [1,2]. There is an association between depression, anxiety and obesity, and several studies have shown that obese women are more vulnerable than obese men to the development of psychiatric and psychological disorders [3-9]. A number of studies have investigated symptoms of depression and anxiety during and after pregnancy [10-13]. Josefsson et al. found in a longitudinal study that the prevalence of depressive symptoms was 17% during late pregnancy and 13% postpartum [10]. Rubertsson et al. found a prevalence of 14% in early pregnancy and 11-14% during the first year postpartum [12]. Both anxiety and depression during pregnancy are strong predictors for postpartum depression [11,13].

Obesity during pregnancy is also associated with a high risk for medical complications [14]. Maternal obesity is, for example, associated with an increased risk for preeclampsia and gestational diabetes mellitus as well as for complications during delivery. In addition, there is an increased risk for antepartum stillbirth and macrosomia [14]. Psychological health and well being during and after pregnancy among obese women have not been thoroughly investigated. The association between body mass index (BMI) and self-reported postpartum depressive symptoms was investigated in a study in the United States [15]. In a stratified random sample of approximately 600 obese women, 30.8% reported moderate or greater depressive symptoms compared with 22.8% in a similar sample consisting of about 1800 normal weight women, two to six months postpartum. Krause and co-workers investigated the prevalence of postpartum depression among approximately 500 overweight and obese postpartum women, recruited from a randomized controlled intervention study designed to encourage postnatal weight loss through increased physical activity and decreased caloric intake [16]. In this study the prevalence of depression was 9.2% and there was no relationship between BMI and postpartum depression.

In a recent prospective intervention study primarily designed to minimize obese women's weight gain to less than 7 kg during pregnancy, we showed that the women who received a structured motivational and behavioral treatment combined with regular physical exercise had a significantly lower weight gain compared with a control group of obese pregnant women who received regular antenatal care without any negative effect on delivery or neonatal outcome [17]. Since it is known that obesity can have a negative influence on psychological well-being, it is important to investigate if the state of psychological well-being of obese pregnant women attending a weight gain restriction program with a focus on behavioral changes differs from psychological well being in a control group. There are to our knowledge no intervention studies designed to accomplish weight-gain restriction for obese pregnant women that have also examined the women's psychological health status.

We hypothesized that pregnant obese women attending an intervention program, based on motivational and behavioral treatment and with the primary aim of reducing weight-gain during pregnancy, would show fewer depressive and anxiety symptoms throughout pregnancy and postnatally than women not attending such a program. Hence, the aim of this study was to investigate psychological well-being measured as symptoms of depression and/or anxiety among obese pregnant women attending a weight gain restriction program and to make comparisons with a control group receiving traditional antenatal care.

## Methods

The Swedish antenatal health care system reaches almost 100% of all pregnant women. The antenatal- and delivery care are free of charge. At the antenatal care clinics (ANC) healthy pregnant women are recommended to attend the regular antenatal program with seven to nine visits to a midwife, and, if needed, arrange for extra appointments with an obstetrician and/or with the midwife.

### Subjects

**Figure 1:**
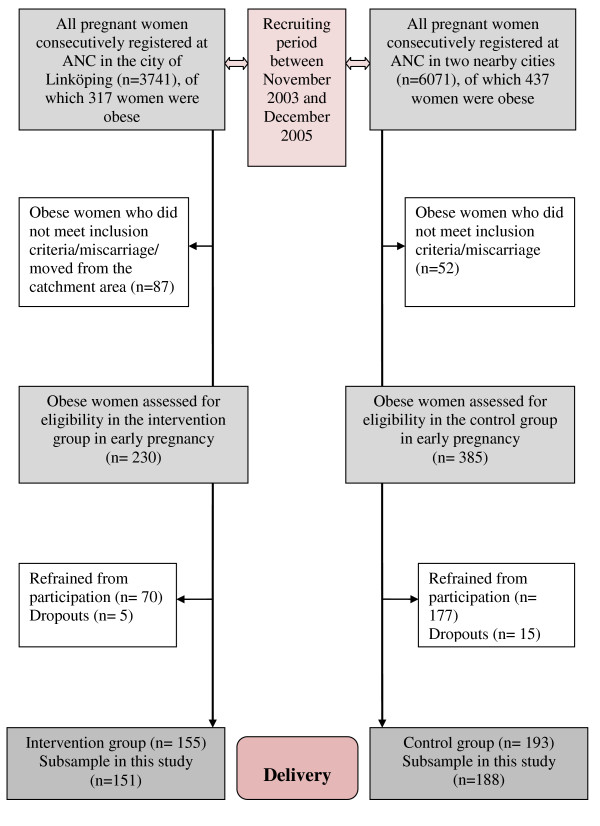
**Description of the study population during the study period**.

During the period November 2003 to December 2005 a total of 317 obese pregnant women in early pregnancy were consecutively registered at the ANC in the city of Linköping and surrounding area. The inclusion criteria for the study were BMI ≥30 and Swedish-speaking. We excluded all women with pre-pregnant diagnosis of diabetes, thyroid dysfunction or a psychiatric disease treated with neuroleptic drugs. After excluding women who did not meet the inclusion criteria, had miscarriage or legal abortion or moved out from the catchment area in early pregnancy, 230 women were eligible and invited to participate. A total of 70 women refrained from participation and five women dropped out during the intervention. One hundred fifty-five women (67.4%) completed the intervention. This subsample consisted of 151 obese women with singleton pregnancies (Figure 1).

All obese pregnant women (n = 437) consecutively registered during the same period at the ANC in two nearby cities with surrounding area, formed a control group. The inclusion- and exclusion criteria were the same as for the intervention women. Thereafter women who had a miscarriage or legal abortion were excluded. Finally, 385 women were eligible and invited to participate. Of this total, 177 women refrained from participation and 15 women dropped out during pregnancy. One hundred ninety-three women (50.1%) completed the participation. This subsample consisted of 188 obese women with singleton pregnancies (Figure 1).

In a previous study, no differences were found between the intervention group and the control group regarding age, parity, marital status, smoking, BMI and occupation [17]. The women in the intervention group reported a higher education level than the women in the control group (p = .044). The women who refrained from participation in both groups were, on average, one year younger than the women who participated in the study (29 vs. 30 years, p = .018). Moreover, those who declined to participate were smokers and had previous children to a higher extent than those participating in the study. For a detailed description of the study participants, see Claesson et al [17].

All data related to pregnancy, delivery and the puerperium were registered in the standardized and identical Swedish antenatal pregnancy, delivery, and neonatal records. The data were manually extracted from the records. This study was approved by the Human Research Ethics Committee, Faculty of Health Sciences, Linköping University.

### Intervention

The obesity intervention program for pregnant women was based on extra visits to a specially trained midwife (author IMC). The women in the intervention group made an average of 22 visits during their pregnancy. The motivational interview/talk followed guidelines set forth by Miller and Rollnick [18]; the goal of this interview was to motivate the obese pregnant woman to change their behavior and to obtain information useful in meeting their needs. The weight gain goal for the study was less than 7 kg and this target was only discussed once during the intervention and that was at the first visit to the midwife. The midwife worked throughout the whole program with assessing the pregnant woman's knowledge of obesity in general and as a risk factor for her pregnancy and delivery outcome as well as for the wellbeing of her child. If the woman lacked sufficient knowledge, she was offered information and given accurate facts. The woman was also informed about the potential consequences of different behaviors associated with eating and food intake; written information was supplied if needed.

All women were given the opportunity to attend an individual 30-min session every week. The session included weight control and counseling characterized by its collaborative structure i.e. counseling based on creating a partnership that honors the woman's expertise and perspectives and enables the counselor to provide an atmosphere that is conducive rather than coercive to change. The woman's own judgment of her motivation and the possibility of changing a behavior, the advantages and disadvantages of changing a behavior, the choice of strategies for adopting and maintaining a new behavior were all topics of the sessions. All women who attended the program were also invited to an aqua aerobic class (once or twice a week), especially designed for obese women. The obese women in the control group attended the routine antenatal care program.

### Measures

The Beck Anxiety Inventory (BAI) was used to measure the severity of anxiety [19]. The BAI consists of a 21-item self-report inventory where each item describes a common symptom of anxiety. The BAI discriminate anxiety from depression [19]. The respondent was asked to rate each symptom over the preceding week on a four point scale (0-3). Scores of 0-7 reflect minimal anxiety, 8-15 mild anxiety, 16-25 moderate anxiety and score of 26-63 indicate severe anxiety [20]. The upper interval limit may be adjusted depending on the purpose for the use. To minimize the rate of false negative results the upper interval limit may decrease and to minimize the rate of false positive results the upper limit may be increased. Some studies have investigated and validated the BAI in non-clinical samples, composed of women as well as men [21-23]. The results support the use of the BAI in a community sample. In this study we used a cut-off level of ≥10 to define symptoms of anxiety.

The Edinburgh Postnatal Depression Scale (EPDS) is a 10-item self-report scale assessing symptoms of depression such as dysphoric mood, anxiety, and feeling of guilt, suicidal ideas and "not coping". Each item is scored on a four point scale (0-3) and rates the intensity of depressive symptoms during the previous 7 days. The scale is specifically designed to screen for postpartum depression but can also be used as a valid measure of dysphoria through the various stages of pregnancy and the puerperium [24]. The validity of the Swedish version has been tested [25]. The EPDS cannot by itself confirm a diagnosis of depressive illness, but when using a cut-off level of >12 Cox et al. [24] showed a sensitivity of 86%, a specificity of 78% and a positive predictive value of 73% for major depressive illness. Another validation of the EPDS by Murray & Carothers [26] also used a cut-off level of >12 showed a sensitivity of 68%, a specificity of 96% and a positive predictive value of 67% for both major and minor depressive illness. To find all actual major depressions, Cox et al. [24] propose a cut-off level ≥10 to reduce detection failure in the postnatal period. When selecting this threshold the sensitivity for detection of major depression increased to almost 100% and the specificity to 82% [27]. In this study we used a cut-off level of ≥10 to define symptoms of depression.

The women in the intervention- and control group answered the BAI and the EDPS at around gestational week 15, 35 and 11 weeks postpartum.

### Statistics

All analyses were performed using the SPSS program, version 16.0 (SPSS Inc., Chicago, IL). Statistical significance was defined as (two-sided) *p *≤ 0.05. Group differences between the intervention women and the control women were estimated by using the χ^2 ^- test on categorical variables. The Student's *t*-test on continuous, normally distributed variables measuring changes in mean score on BAI and EPDS over time was used. Analyses were performed between the intervention- and control group as well as within the groups. Furthermore, to make a more comprehensive assessment of group differences, between as well as within the two groups, logistic regressions were performed with the three BAI and EPDS measurements as dependent variables. The grouping variable has been adjusted for socio-demographic variables (age, parity, marital status, socioeconomic factors and occupational status) and complications during pregnancy (hyperemesis, gestational diabetes mellitus, preeclampsia, premature contractions, lumbar and pelvic pain), complications during delivery (acute cesarean section, instrumental delivery, induced delivery, bleeding >1000 ml, perineal tears) and neonatal complications (small for gestational age, preterm <37 weeks, Apgar Score at 5 min <7 and Apgar Score at 10 min <7).

## Results

### Anxiety symptoms

In analyses of the anxiety symptoms using the BAI, no significant differences in the prevalence of symptoms were found between the groups (Table 1).

**Table 1:** Symptoms of anxiety at gestational week 15 and 35, and 11 weeks postpartum.

		Intervention group		Control group		
	**Anxiety symptoms**	**n**	**%**	**n**	**%**	P*

**Gestational week 15**						.719

	Absent**	114	75.5	142	77.2	

	Present***	37	24.5	42	22.8	

**Gestational week 35**						.648

	Absent**	108	76.1	127	78.4	

	Present***	34	23.9	35	21.6	

**Postpartum**						.557

	Absent**	130	90.9	127	88.8	

	Present***	13	9.1	16	11.2	

* X^2 ^test

** Beck Anxiety Inventory (BAI) score <10

*** Beck Anxiety Inventory (BAI) score ≥10

Bivariate analysis showed that there was a difference within the intervention group due to socioeconomic factors at the time of the two measurements during pregnancy (p = .045 and p = .000). Women with a lower level of education had symptoms of anxiety more often than the women who had a higher level of education. The same results were found within the control group (p = .010 and p = .012). There was also a difference within the control group at the time of the two measurements during pregnancy concerning occupational status (p = .003 and p = .009). Women who were unemployed showed symptoms of anxiety more often than women who were gainfully employed. The multivariate analyses (logistic regression) showed no difference between the intervention group and the control group after adjustment for socio-demographic variables and pregnancy- and delivery complications (Table 2).

**Table 2:** Symptoms of anxiety† during pregnancy and postpartum in relation to socio-demographic factors and pregnancy- and obstetric complications

		Gestational week 15				Gestational week 35				11 weeks postpartum			
				95% CI* for OR**				95% CI* for OR**				95% CI *for OR**	
		p***	OR	Lower	Upper	p***	OR	Lower	Upper	p***	OR	Lower	Upper
Group	Women in the intervention group	Reference level				Reference level				Reference level			
	Women in the control group	.399	.789	.454	1.369	.326	.739	.404	1.351	.651	1.213	.525	2.800
													
Parity	Primipara	Reference level				Reference level				Reference level			
	Multipara	.805	.932	.534	1.628	.547	.829	.451	1.525	.283	1.618	.673	3.894
													
Smoking	No	Reference level				Reference level				Reference level			
	Yes	.345	1.543	.627	3.795	.544	1.380	.488	3.896	.059	3.153	.956	10.403
													
Occupation	Unemployed	Reference level				Reference level				Reference level			
	Unskilled	.302	.570	.196	1.657	.045	.284	.083	.973	.645	1.562	.234	10.410
	Students/others	.800	.881	.330	2.350	.460	1.498	.513	4.377	.293	2.578	.442	15.048
	Skilled/lower white collar workers	.064	.382	.138	1.056	.010	.217	.068	.696	.755	1.338	.214	8.363
	Middle/high white collar workers	.024	.239	.069	.826	.008	.160	.041	.622	.926	1.102	.144	8.409
													
Employment	Gainfully employed	Reference level				Reference level				Reference level			
	Not gainfully employed	.224	1.597	.751	3.397	.928	.961	.399	2.312	.656	1.281	.431	3.808
													
Pregnancy complication	No	Reference level				Reference level				Reference level			
	Yes	.014	1.983	1.147	3.430	.009	2.238	1.221	4.102	.664	1.202	.523	2.761
													
Delivery complication	No	Reference level				Reference level				Reference level			
	Yes	.661	1.129	.656	1.944	.011	.436	.230	.824	.143	.499	.197	1.265

†Defined as score ≥10 in measurements with Beck Anxiety Inventory

* CI = Confidence Interval ** OR = Odds Ratio

***Statistical significance defined as *p *≤ 0.05

Measurements of fluctuations in symptoms of anxiety between the two assessment points in gestational week 15 and 11 weeks postpartum showed no differences either within or between the intervention group and the control group. A total of 5% of women in the intervention group and 4% of women in the control group had symptoms of anxiety at all three assessment points. As shown in Table 3, there was no difference in symptoms of anxiety in relation to weight gain between the intervention group and the control group at the time of the different assessments. Neither was there any difference between the occurrence of anxiety symptoms within the intervention and within the control group (data not shown).

**Table 3:** Symptoms of anxiety in relation to weight gain at gestational week 15 and 35 and 11 weeks postpartum.

		Weight gain during pregnancy <7 kg					Weight gain during pregnancy >7 kg				
		**Intervention group**		**Control group**			**Intervention group**		**Control group**		

	**Anxiety symptoms**	**n**	**%**	**n**	**%**	p*	**n**	**%**	**n**	**%**	p*

**Gestational week 15**						.441					.888

	Absent**	39	76.5	25	80.6		69	76.7	96	77.4	

	Present***	12	23.5	6	19.4		21	23.3	28	22.6	

**Gestational week 35**						.688					.376

	Absent**	34	75.6	21	77.8		68	77.3	87	78.4	

	Present***	11	24.4	6	22.2		20	22.7	24	21.6	

**Postpartum**						.309					.877

	Absent**	45	93.8	21	87.5		78	91.8	90	90.0	

	Present***	3	6.2	3	12.5		7	8.2	10	10.0	

* X^2 ^test

** Beck Anxiety Inventory (BAI) score <10

*** Beck Anxiety Inventory (BAI) score ≥10

### Depressive symptoms

There were no differences in the occurrence of symptoms of depression between the groups (Table 4).

**Table 4:** Depressive symptoms at gestational week 15 and 35 and 11 weeks postpartum.

		Intervention group		Control group		
	**Depressive symptoms**	**n**	**%**	**n**	**%**	p*

**Gestational week 15**						.882

	Absent**	122	81.3	150	82.0	

	Present***	28	18.7	33	18.0	

**Gestational week 35**						.341

	Absent**	110	78.0	132	82.5	

	Present***	31	22.0	28	17.5	

**Postpartum**						.849

	Absent**	127	88.8	128	89.5	

	Present***	16	11.2	15	10.5	

* X^2 ^test

** Edinburgh Postnatal Depression Scale (EPDS) score <10

*** Edinburgh Postnatal Depression Scale (EPDS) score ≥10

A total of 18.7% of women in the intervention group and 18.0% in the control group showed depressive symptoms in early pregnancy (Table 4). In late pregnancy, the percentage of women with depressive symptoms was found to be 22.0% in the intervention group and 17.5% in the control group. Postpartum the percentages were 11.2% in the intervention group and 10.5% in the control group.

Bivariate analyses showed that a lower level of education was related to symptoms of depression in the intervention group in gestational week 15 (p = .002) and in gestational week 35 (p = .003). Similar results were found in the control group in gestational week 35 (p = .001). There was also a relationship between occupational status and depressive symptoms: in the intervention group in gestational week 15 (p = .010), in the control group in gestational week 35 (p = .001) and 11 weeks postpartum in both groups (p = .049 respectively p = .017). Women who were unemployed showed more symptoms of depression than women who were gainfully employed. Analyses within the groups showed a significant relation in the control group between complications during pregnancy; i.e. diabetes, preeclampsia, preterm contractions etc. and symptoms of depression (p = .010). No such relationship could be found in the intervention group. The multivariate analyses (logistic regression) show that there was no difference between the intervention group and the control group after adjustment for socio-demographic variables and pregnancy- and delivery complications (Table 5).

**Table 5:** Symptoms of depression† during pregnancy and postpartum in relation to socio-demographic factors and pregnancy- and obstetric complications

		Gestational week 15				Gestational week 35				11 weeks postpartum			
				95% CI* for OR**				95% CI* for OR**				95% CI* for OR**	
		p***	OR	Lower	Upper	p***	OR	Lower	Upper	p***	OR	Lower	Upper
Group	Women in the intervention group	Reference level				Reference level				Reference level			
	Women in the control group	.896	1.055	.469	2.376	.363	.673	.287	1.578	.650	1.235	.497	3.071
													
Parity	Primipara	Reference level				Reference level				Reference level			
	Multipara	.022	2.825	1.160	6.880	.344	1.522	.638	3.634	.341	1.601	.607	4.225
													
Smoking	No	Reference level				Reference level				Reference level			
	Yes	.077	2.722	.897	8.259	.070	3.038	.913	10.106	.111	2.882	.785	10.581
													
Occupation	Unemployed	Reference level				Reference level				Reference level			
	Unskilled	.893	.909	.229	3.615	.174	.320	.062	1.650	.934	1.078	.184	6.328
	Students/others	.793	1.178	.347	4.000	.393	.535	.127	2.248	.503	1.707	.357	8.172
	Skilled/lower white collar workers	.053	.237	.055	1.019	.007	.093	.017	.518	.840	.839	.154	4.567
	Middle/high white collar workers	.102	.208	.032	1.362	.031	.121	.018	.821	.856	.836	.120	5.805
													
Employment	Gainfully employed	Reference level				Reference level				Reference level			
	Not gainfully employed	.282	1.781	.622	5.105	.460	.622	.176	2.193	.198	2.110	.677	6.582
													
Pregnancy complication	No	Reference level				Reference level				Reference level			
	Yes	.018	2.682	1.188	6.056	.003	3.825	1.576	9.285	.451	1.416	.574	3.494
													
Delivery complication	No	Reference level				Reference level				Reference level			
	Yes	.696	.850	.377	1.919	.994	.997	.428	2.320	.745	.856	.334	2.193

†Defined as score ≥10 in measurements with Edinburgh Postnatal Depression Scale

* CI = Confidence Interval ** OR = Odds Ratio

***Statistical significance defined as *p *≤ 0.05

Measurements of fluctuations in depressive symptoms at the assessment points in gestational week 15 and 11 weeks postpartum showed no differences either within or between the intervention group and the control group. A total of 6% of women in the intervention group and 4% of women in the control group had symptoms of depression at all three assessments. As shown in Table 6, there was no difference in symptoms of depression in relation to weight gain in the intervention group and the control group at the time of the different assessments. Neither was there any difference between the occurrence of depressive symptoms within the intervention group and the control group (data not shown).

**Table 6:** Depressive symptoms in relation to weight gain at gestational week 15 and 35 and 11 weeks post partum.

		Weight gain during pregnancy <7 kg					Weight gain during pregnancy >7 kg				
		**Intervention group**		**Control group**			**Intervention group**		**Control group**		

	**Depressive symptoms**	**n**	**%**	**n**	**%**	p*	**n**	**%**	**n**	**%**	p*

**Gestational week 15**						.378					.688

	Absent**	45	88.2	26	81.2		71	79.8	100	82.0	

	Present***	6	11.8	6	18.8		18	20.2	22	18.0	

**Gestational week 35**						.153					.596

	Absent**	33	75.0	24	88.9		73	83.0	88	80.0	

	Present***	11	25.0	3	11.1		15	17.0	22	20.0	

**Postpartum**						.366					.387

	Absent**	45	93.8	21	87.5		76	89.4	93	93.0	

	Present***	3	6.2	3	12.5		9	10.6	7	7.0	

* X^2 ^test

** Edinburgh Postnatal Depression Scale (EPDS) score <10

*** Edinburgh Postnatal Depression Scale (EPDS) score ≥10

A total of six women in the intervention group (4.0%) and three women in the control group (1.6%) had symptoms of both anxiety and depression at all three assessment points.

## Discussion

In this prospective intervention study with a primary aim of minimizing gestational weight gain of obese women, the prevalence of symptoms of anxiety and depression during pregnancy varied between 18% and 25% within the intervention- and control group. The postnatal prevalence of symptoms of anxiety and depression varied between 9% and 11% within these two groups. No differences were found between the groups. Around 4-5% of all women had symptoms of anxiety, 4-6% had symptoms of depression and only a few women presented symptoms of both anxiety and depression at all three assessments. There was no relationship between symptoms of anxiety, depression and weight gain during pregnancy as measured on three different occasions. In both groups, women with a lower level of education and without employment showed symptoms of anxiety and depression more often than women with a higher level of education and gainful employment. After adjustment for socio-demographic variables and pregnancy, delivery and neonatal complications there were no differences in the prevalence of symptoms of anxiety between the groups. However, there was a relation between complications during pregnancy and symptoms of depression in the control group.

We hypothesized that participation in the intervention program would increase psychological well-being due to weekly motivational support and would therefore result in a lower prevalence of anxiety or depressive symptoms than would have resulted without the program. This hypothesis was not confirmed. Neither did we find any relation between weight gain during pregnancy or postnatal weight and the prevalence of symptoms of anxiety or depression among obese women in the intervention group and the control group. One can consider whether socioeconomic factors such as unemployment and or a low level of education compose a stress on the individual and the effect this has. It is possible, as suggested by Britton [28], that mothers with a low level of education may have a tendency towards developing high levels of anxiety because they are less able to handle the demands and expectations placed on them during the period when they take on the maternal role. One may also speculate if worrying about the future, as concerns both employment and household economy, may lead to symptoms of both anxiety and depression.

The prevalence of anxiety in a general population of pregnant women during and after pregnancy has been investigated in earlier studies [13,29,30]. In our study the prevalence of symptoms of anxiety among obese pregnant women was around 23% during pregnancy, which is in accordance with findings by other authors [13,30]. Breitkopf et al. assessed anxiety symptoms among pregnant, non-pregnant and postnatal women, and found that the anxiety scores were lower among postnatal women in comparison with pregnant and non-pregnant women even after controlling for depressive symptoms [29]. This is in line with our results where the symptoms of anxiety were lower postpartum than during pregnancy. Nothing in our study indicates that obese pregnant or postnatal women have more symptoms of anxiety than are exhibited in the general pregnant population.

Several studies have investigated the prevalence of depressive symptoms during pregnancy and postpartum [10,12,31-34]. The EDPS has been used with different cut-offs for evaluating depressive symptoms [10,12,31,33,34]. The prevalence in these studies varies from 8% to 17% during pregnancy and 9% to 13% postpartum. A study that used the same cut-off on the EPDS for depressive symptoms as in this study found a prevalence of depressive symptoms during late pregnancy and postpartum similar to what we found [10].

Furthermore, two studies have investigated the impact of obesity on the risk for postpartum depression with differing results [15,16]. In the study of LaCoursiere the woman was asked to assess her depressive symptoms' two - six months after delivery [15]. The five-level scale was from "not depressed at all" to "very depressed and had to get help". Answer on symptoms' level 'moderately' or more, indicated postnatal self-reported depression and was given by 31% of the obese women postpartum [15]. In the study of Krause et al., where the women completed the EPDS with a cut off of ≥13, six weeks postpartum, a prevalence of 9% among overweight and obese women postpartum was shown [16]. Our results at the postnatal measurement were in accordance with the findings in the study by Krause.

The relationship between antenatal and postnatal symptoms of depression has been investigated by Josefsson et al. who showed that 6% of the women had symptoms both during pregnancy and postpartum and this is in line with our findings [10]. The occurrence of depressive symptoms during and after pregnancy in relation to demographic characteristics in a general pregnant population has also been investigated [12,16,32,33,35]. These studies have shown an association between economic difficulties, low household income, unemployment, lower educational attainment and depressive symptoms [12,16,32,33]. The connection between anxiety and depressive symptoms and maternal and neonatal outcome has been investigated [36-38]. A review by Alder and colleagues found elevated levels of anxiety and depression to be related to obstetric complications, preterm labor and alleviation of labor pain [36]. Vollebregt et al. investigated the association of preeclampsia and gestational hypertension with psychosocial stress among nulliparous in the first half of pregnancy and found that anxiety, pregnancy-related anxiety or depression had no effect on the incidence of preeclampsia and gestational hypertension [38]. Berle and co-authors reported a relationship between anxiety disorder during pregnancy and low Apgar score at one and five minutes, but no relationship was observed with low birth weight or preterm delivery [37]. We did not find any differences between the two groups of women in our study regarding the relationship between symptoms of anxiety and/or depression and pregnancy-, delivery- and neonatal complications. In the control group, however, there was a relationship between pregnancy complications and depressive symptoms at the assessment in late pregnancy.

This study was not randomized, which can be seen as an important limitation. In all scientific research it is important to control external factors and the environment has been found to exert a powerful influence on people's emotions and behavior [39] and careful consideration must be given to ensure that the intervention group and the control group will get treatment and care at the same setting. We chose therefore to use ANCs in two nearby cities to serve as controls. The antenatal programs in Sweden are standardized and almost identical concerning the management of the pregnancy, which ensures similar care at different ANCs. We were also able to control for several background characteristics that otherwise could confound the results. There was also a difference in the completion rates between the intervention- and control group. A total of five women in the intervention group dropped out compared with 15 women in the control group. Another limitation is that the number of questionnaires answered at the times of the three assessments differs to some extent between the intervention- and control group. Therefore some caution is advisable when generalizing these results. Furthermore, there are no data on prevalence of symptoms of anxiety and depression among women who declined participation. Despite these limitations there are some important findings in this study. To our knowledge this is the first intervention study that has investigated symptoms of anxiety and/or depression in an obese pregnant population. Since there are few studies that have investigated the relation between anxiety and depression among obese pregnant women especially in relation to a weight gain intervention program there is need for further work on this topic.

## Conclusions

In conclusion, neither weight gain nor a weight gain restriction program during pregnancy seems to influence the prevalence of symptoms of anxiety or depression. Obese women who participated in this study do not run a higher risk for anxiety and/or depressive symptoms during pregnancy or postpartum, compared with a general pregnant and postnatal population.

## Competing interests

The authors declare that they have no competing interests.

## Authors' contributions

I-M C contributed to the design of this study, collection and analysis of the data, and preparation of the manuscript.

AJ and GS contributed to research idea, design and preparation of the manuscript.

All authors have read and approved the final version of the manuscript.

## Pre-publication history

The pre-publication history for this paper can be accessed here:

http://www.biomedcentral.com/1471-2458/10/766/prepub
